# Comparison of Machine Learning Models and the FMF Competing-Risks Algorithm for First-Trimester Preeclampsia Screening in a Romanian Cohort

**DOI:** 10.3390/diagnostics16101540

**Published:** 2026-05-19

**Authors:** Alexandra-Elena Cristofor, Alexandru Carauleanu, Ingrid-Andrada Vasilache, Iustina Condriuc, Ovidiu Bica, Dragos Nemescu

**Affiliations:** 1 Grigore T. Popa University of Medicine and Pharmacy, 700115 Iasi, Romaniaiustina_condriuc@yahoo.com (I.C.);; 2 Faculty of Medicine and Biological Sciences, ‘Ștefan cel Mare’ University, 720229 Suceava, Romania

**Keywords:** preeclampsia, first trimester screening, Bayes theorem, logistic regression, random forest, XGBoost

## Abstract

**Background/Objectives**: First-trimester preeclampsia (PE) screening is most widely implemented using the Fetal Medicine Foundation (FMF) algorithm, which combines maternal factors with biophysical and biochemical markers via a competing-risks/Bayes framework to produce individualized risks and guide prophylaxis decisions. We aimed to compare commonly used machine-learning (ML) classifiers (logistic regression, random forest, XGBoost) against FMF a priori and a posteriori risk estimates in a Romanian screening cohort. **Methods**: We analyzed 1583 singleton pregnancies screened at 11–14 weeks’ gestation. Primary analyses excluded aspirin-treated women to reduce treatment-induced outcome modification. We evaluated two feature sets mirroring FMF structure: (1) a maternal-factor “a priori” set and (2) a “a posteriori” set additionally incorporating mean arterial pressure (MAP), uterine artery pulsatility index (UtA-PI), and Pregnancy-Associated Plasma Protein A (PAPP-A). Models were trained using stratified repeated cross-validation (5-fold × 10 repeats) and evaluated using AUC-ROC, DeLong tests, and sensitivity at 10% false-positive rate. Calibration of the model, sensitivity analyses and decision-curve analysis (DCA) were also assessed. **Results**: In the a priori comparison, the best ML model was logistic regression (AUC 0.796) versus FMF prior risk AUC 0.841 (DeLong *p* = 0.349). The sensitivity at 10% false positive rate (FPR) was 33.3% for the model versus 50.0% for FMF model. In the a posteriori comparison, the best ML model was random forest (AUC 0.844) versus FMF posterior risk AUC 0.929 (DeLong *p* = 0.087), with sensitivity at 10% FPR of 57.1% for ML and 71.4% for FMF. Random undersampling did not improve ML performance. Including aspirin-treated pregnancies did not significantly change our results. **Conclusions**: In this study, the FMF competing-risks outputs outperformed or matched ML classifiers in both maternal-only and biomarker-augmented screening, and DCA favored FMF particularly for the a posteriori model.

## 1. Introduction

Preeclampsia remains a major contributor to maternal and perinatal morbidity, and early identification of women at elevated risk is central to prevention strategies. Contemporary first-trimester screening aims to identify high-risk patients early enough to implement risk-reducing interventions, with the Fetal Medicine Foundation (FMF) approach now widely used worldwide.

The FMF competing-risks framework generates patient-specific risk by combining a prior distribution derived from maternal characteristics with likelihood functions from biophysical and biochemical markers using Bayes’ theorem, thus producing individualized posterior risks for preeclampsia before specified gestational-age thresholds [1]. In the original large cohort of 35,948 singleton pregnancies, combined screening with maternal factors, mean arterial pressure (MAP), uterine artery pulsatility index (UtA-PI) and placental growth factor (PlGF) predicted 75% (95% CI 70–80%) of preterm PE (<37 weeks) and 47% (95% CI 44–51%) of term PE, at a fixed 10% false-positive rate (FPR), whereas maternal factors alone detected 49% (95% CI 43–55%) of preterm and 38% (95% CI 34–41%) of term PE [2].

Subsequent multicenter validation in eight units in Spain (10,110 pregnancies) reported a detection rate of 72.7% (95% CI 62.9–82.6%) for preterm PE at a 10% screen-positive rate when combining maternal characteristics, MAP, UtA-PI and PlGF [3,4]. External validations in Australia (29,609 pregnancies) and China (10,899 pregnancies) found that combined screening using maternal factors, MAP, UtA-PI and biochemical markers detected 71% (95% CI 59–84%) and 65.0% of preterm PE at 10% FPR, respectively, with detection increasing to 72.7% and 76.1% as FPR rose to 15% and 20% in the Chinese cohort [5,6]. Consistent with these data, review articles summarize that the FMF triple test achieves detection rates of about 90% for early PE (<34 weeks) and 75% for preterm PE (<37 weeks) at 10% FPR, markedly outperforming approaches based on maternal risk factors alone [7,8,9].

Machine learning (ML) methods, especially ensemble techniques such as random forest (RF) and extreme gradient boosting (XGBoost), have shown strong predictive performance for preeclampsia, often achieving higher area under the curve (AUC) values than classical regression models. Systematic reviews report that random forest and XGBoost can reach AUCs up to 0.94 and 0.92, respectively, outperforming many traditional models, although heterogeneity across studies and limited external validations remain challenging [10,11,12]. In a large validation cohort of 12,549 pregnancies, XGBoost and logistic regression showed similar discrimination with AUCs of 0.75 (95% CI 0.73–0.78) and 0.76 (95% CI 0.74–0.78), respectively, while random forest had a slightly lower AUC of 0.71 (95% CI 0.69–0.74). Also, logistic regression demonstrated superior calibration compared to ML models [13].

Comparisons between ML models and the FMF competing-risks model are complicated by differences in biomarker quality, population-specific marker distributions, and assay harmonization. An ML model using the same predictors as FMF (maternal factors, MAP, UtA-PI, PlGF) initially performed significantly worse than FMF until retrained with adjustments for PlGF analyzer differences. After retraining, AUCs were comparable for preterm and term PE prediction but remained inferior for any PE [14]. Overall, while ML ensemble methods show promise with high discrimination metrics, simpler models like logistic regression or well-calibrated competing-risks models remain critical for clinical deployment due to their better calibration and interpretability.

In Romania and other countries where the completeness of biochemical markers can be limited, it remains unclear whether commonly deployed ML classifiers trained on locally available data can match the discriminative and clinical-utility performance of FMF risk estimates and whether ML may offer advantages when some biomarkers (notably PlGF) are missing or sparse. We therefore conducted a comparison of ML classifiers (logistic regression, random forest, XGBoost) against FMF a priori and a posteriori risks in a Romanian first-trimester PE screening cohort.

## 2. Materials and Methods

### 2.1. Study Design and Population

This retrospective analysis used a database of pregnancies screened at 11–14 weeks’ gestation in a single center. The primary cohort comprised 1583 screened pregnancies, and the prespecified primary analysis excluded aspirin-treated patients (n = 211) to mitigate treatment-related outcome modification because aspirin prophylaxis can influence downstream disease incidence and therefore bias apparent screening performance metrics in “screen-and-treat” settings. A secondary analysis repeated the evaluation, including aspirin-treated pregnancies.

Preeclampsia was defined as the presence of a recorded diagnosis or onset information in any PE-specific clinical field (PE status, gestational age at onset, or recorded preeclampsia diagnosis). The diagnosis was coded as binary.

### 2.2. FMF Risk Outputs

FMF risk estimates were calculated as (i) prior (a priori) risk and (ii) posterior (a posteriori) risk, reflecting the FMF Bayes-theorem approach that combines a prior distribution from maternal factors with likelihoods from biomarkers to produce patient-specific posterior risks.

### 2.3. Predictors and Feature Sets

Two feature sets were evaluated, designed to align conceptually with FMF’s a priori versus a posteriori structure:

- A priori (maternal factors): maternal age, body mass index (BMI), previous pregnancies, method of conception, smoking, diabetes, chronic hypertension, maternal family history of PE, and multiparity.
- A posteriori (maternal factors + biomarkers): a priori predictors plus MAP (mean arterial pressure), UtA-PI, and PAPP-A MoM.

### 2.4. Missing Data Handling

Missingness was quantified for all biomarkers and FMF-derived risk variables. The primary machine-learning analyses used a complete-case approach. To assess the potential impact of missingness, participants included in the complete-case a posteriori analyses were compared with excluded participants using Mann–Whitney U tests for continuous variables and chi-square or Fisher exact tests for categorical variables, as appropriate.

As a sensitivity analysis, multiple imputation using iterative imputation with posterior sampling was performed for retained predictors. Outcome status and FMF risk estimates were not imputed.

### 2.5. Machine-Learning Models

We trained using Python software (version 3.12.13, Python Software Foundation, Beaverton, OR, USA) three ML classifiers: logistic regression, RF, and XGBoost for PE prediction. The selected machine-learning algorithms were chosen to represent complementary modeling approaches commonly used in clinical prediction research and to allow comparison between interpretable linear methods and more flexible non-linear ensemble methods. These analyses were considered exploratory because of the very small number of preeclampsia events and the consequent high risk of overfitting.

Logistic regression was fitted with a maximum of 5000 iterations. Random forest models were trained using 500 trees, square-root feature selection at each split, a minimum leaf size of 5, and class weighting to account for the low event rate. XGBoost models were trained with 200 estimators, a maximum tree depth of 4, a learning rate of 0.1, subsampling of 0.8, column sampling of 0.8, a minimum child weight of 5, and a scale-pos-weight parameter calculated from the non-event to event ratio. Because the number of PE events was low relative to the number of predictors, the machine-learning analyses were considered exploratory and interpreted cautiously because of the increased risk of model overfitting and optimistic performance estimation despite repeated cross-validation.

### 2.6. Cross-Validation and Prediction Generation

We used stratified repeated k-fold cross-validation (5 folds × 10 repeats) to reduce variance in performance estimates. For each ML model, we generated out-of-fold predicted probabilities across all repeats and aggregated them per observation to produce a single cross-validated predicted probability per individual.

### 2.7. Performance Measures

We evaluated discrimination using AUC-ROC and compared AUCs between ML and FMF using DeLong tests. Screening performance was summarized using sensitivity at 90% specificity (equivalently 10% FPR), reflecting common reporting in FMF validations and the clinical context of fixed screen-positive rates. We additionally examined sensitivity, specificity, PPV, and NPV at a clinically interpretable FMF threshold (1:100), consistent with the literature.

### 2.8. Calibration and Decision-Curve Analysis

We assessed calibration using decile-based calibration plots comparing predicted risk to observed outcome frequency. Clinical net benefit was evaluated via decision-curve analysis (DCA).

### 2.9. Feature Attribution

For the a posteriori XGBoost model, we computed SHAP values to quantify feature contributions to individual predictions and summarize global importance.

### 2.10. Sensitivity Analysis with Aspirin

We repeated the primary analyses, including aspirin-treated pregnancies, because aspirin can alter PE incidence and influence apparent screening performance.

### 2.11. Sensitivity Analysis with Random Undersampling

Because our primary cohort had low PE prevalence, we conducted a random undersampling analysis to test whether class imbalance was responsible for ML underperformance. In each setting (a priori and a posteriori), we undersampled the majority class (non-PE) to achieve PE:non-PE ratios of 1:5 and 1:10. Each undersampling ratio was repeated 10 times using different random seeds, and model performance was summarized by the median AUC and interquartile range (IQR) across repetitions. Cross-validation splits were adapted to ensure feasibility given the reduced number of PE cases.

### 2.12. Multiple Imputation Sensitivity Analysis

As a sensitivity analysis, multiple imputation was performed for predictors retained in the actual models. Outcome status and FMF risk estimates were not imputed.

Multiple imputation was performed using iterative imputation with posterior sampling. Twenty imputed datasets were generated. For each imputed dataset, the same exploratory machine-learning modeling strategy was repeated, and AUC estimates were summarized using the mean, standard deviation, median, and interquartile range across imputations.

## 3. Results

### 3.1. Cohort Characteristics

The full cohort comprised 1583 singleton pregnancies screened at 11–14 weeks of gestation, of which 15 developed preeclampsia (0.95%). After exclusion of aspirin-treated pregnancies, 1372 pregnancies remained, with 12 PE events (0.87%). The a posteriori complete-case cohort included 901 pregnancies with 7 PE events (0.78%).

Patients who developed preeclampsia had higher body mass index (median 24.7 vs. 22.1 kg/m^2^, *p* = 0.004), higher mean arterial pressure (median 93.4 vs. 88.7 mmHg, *p* = 0.016), and were more likely to have chronic hypertension (25.0% vs. 1.5%, *p* < 0.001). Uterine artery pulsatility index tended to be higher (median 1.9 vs. 1.6, *p* = 0.08), and PAPP-A levels tended to be lower (median 0.5 vs. 1.0 MoM, *p* = 0.09) in affected pregnancies, though neither reached conventional statistical significance (Table 1).

### 3.2. Missing Data

Table 2 compares participants included in the a posteriori complete-case analysis with those excluded because of missing biomarker or predictor data. Overall, 901 participants with complete data were included in the final a posteriori analysis, whereas 471 participants were excluded due to incomplete information.

The prevalence of preeclampsia did not differ significantly between included and excluded participants (7/901 [0.8%] vs. 5/471 [1.1%], *p* = 0.8163), suggesting that exclusion due to missing data did not substantially alter the distribution of the primary outcome.

However, several maternal and biomarker-related variables differed significantly between groups. Compared with included participants, excluded participants were slightly younger (median age 29.5 vs. 30.2 years, *p* = 0.0427), had lower mean arterial pressure (87.84 vs. 89.00 mmHg, *p* = 0.0305), higher uterine artery pulsatility index values (1.66 vs. 1.57, *p* = 0.0014), and lower PAPP-A concentrations (0.66 vs. 0.97 MoM, *p* = 0.0275). In addition, both prior FMF risk estimates (*p* = 0.0010) and posterior FMF risk estimates (*p* < 0.001) differed significantly between included and excluded participants.

### 3.3. A Priori and a Posteriori Model Comparison

**Figure 1:**
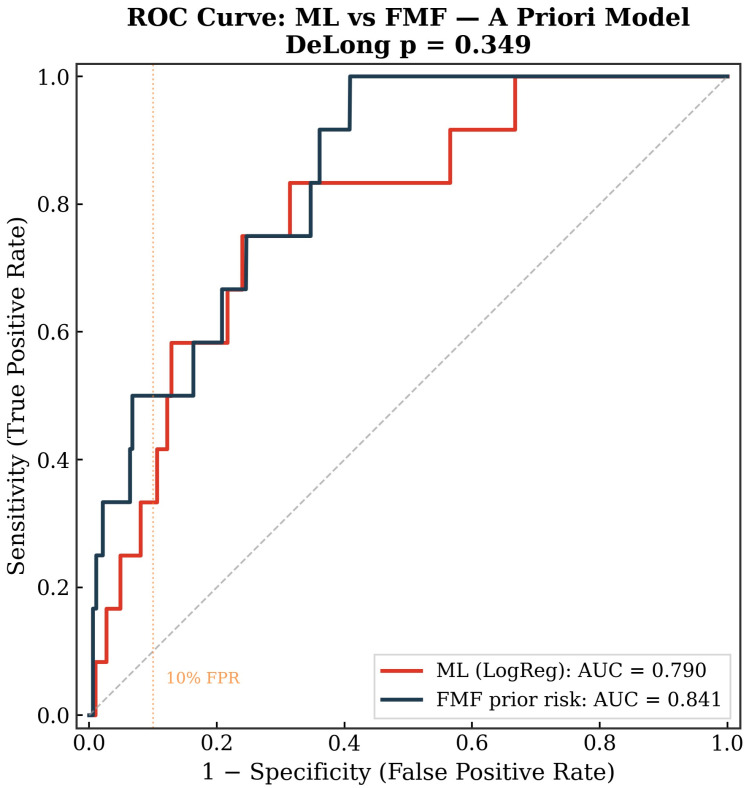
Receiver operating characteristic (ROC) curves comparing the best-performing machine-learning model (logistic regression) versus the FMF a priori risk estimate for the prediction of preeclampsia.

Using maternal characteristics alone (age, BMI, parity, conception method, smoking, diabetes, chronic hypertension, family history of preeclampsia, and twin gestation), logistic regression was the best-performing ML algorithm with a cross-validated AUC of 0.80 (±0.11)—Table 3. This was numerically lower than the FMF prior risk model (AUC 0.84), though the difference was not statistically significant by the DeLong test (*p* = 0.34)—Figure 1. At a fixed 10% false-positive rate, the FMF model detected 50% of preeclampsia cases compared with 33% for the ML model.

**Figure 2:**
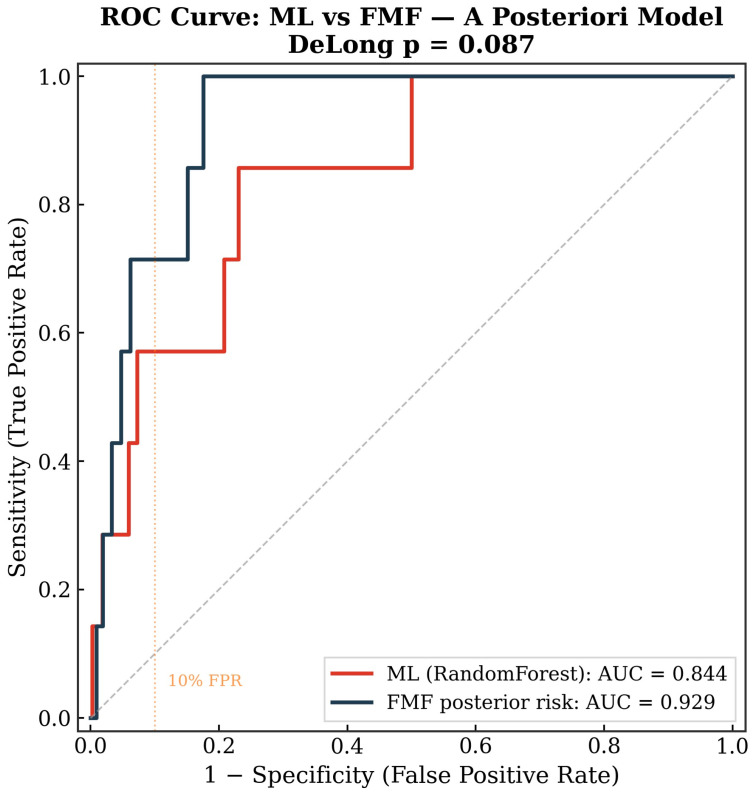
ROC curves comparing the best-performing machine-learning model (random forest) versus the FMF a posteriori risk estimate for the prediction of preeclampsia.

When mean arterial pressure, uterine artery pulsatility index, and PAPP-A were added to the maternal factors, the best-performing ML algorithm was random forest (cross-validated AUC 0.85 ± 0.14)—Table 2, Figure 2. The FMF posterior model achieved an AUC of 0.93 but did not reach statistical significance (DeLong *p* = 0.087; bootstrap 95% CI: ML 0.70–0.97 vs. FMF 0.88–0.98). At a 10% false-positive rate, detection rates were 57% for the ML model and 71% for FMF.

### 3.4. Incremental Biomarker Analysis

**Figure 3:**
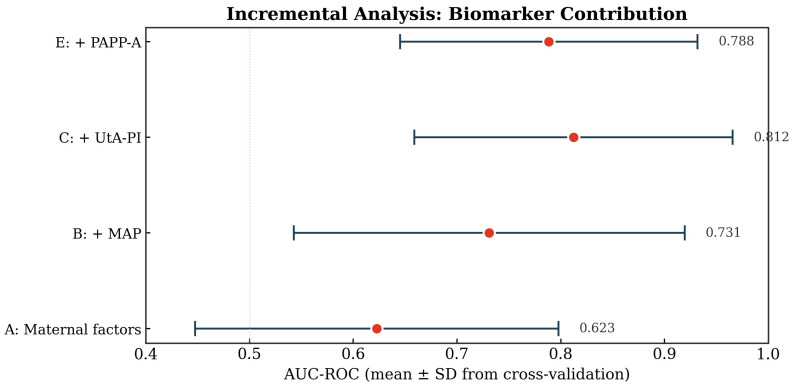
Incremental contribution of biomarkers to discrimination (AUC) for an XGBoost model.

Incremental addition of biomarkers to the XGBoost model demonstrated progressive improvement in discrimination: maternal factors alone yielded an AUC of 0.62, which increased to 0.73 with the addition of MAP, and to 0.81 with further addition of uterine artery pulsatility index. The subsequent addition of PAPP-A did not improve performance (AUC 0.79)—Figure 3.

### 3.5. Feature Importance and SHAP Analysis

**Figure 4:**
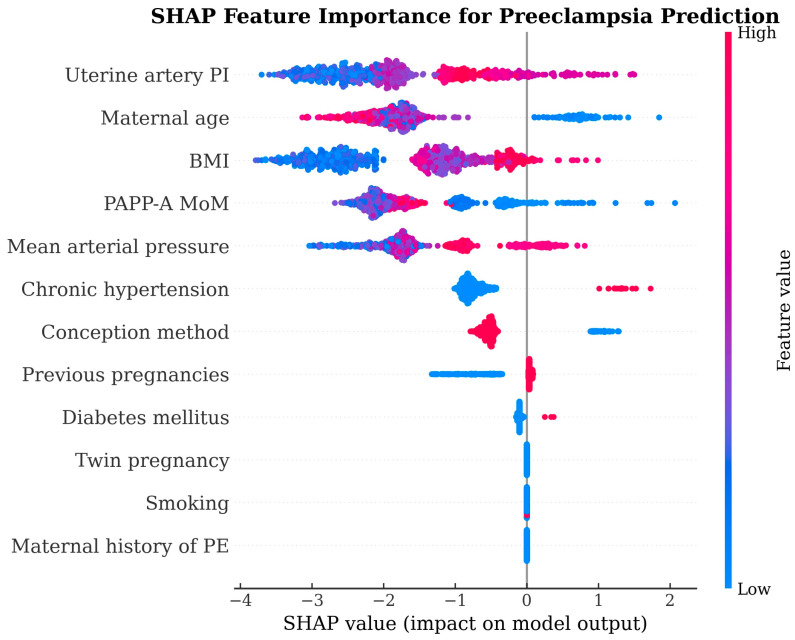
SHAP summary plot for the XGBoost a posteriori model.

**Figure 5:**
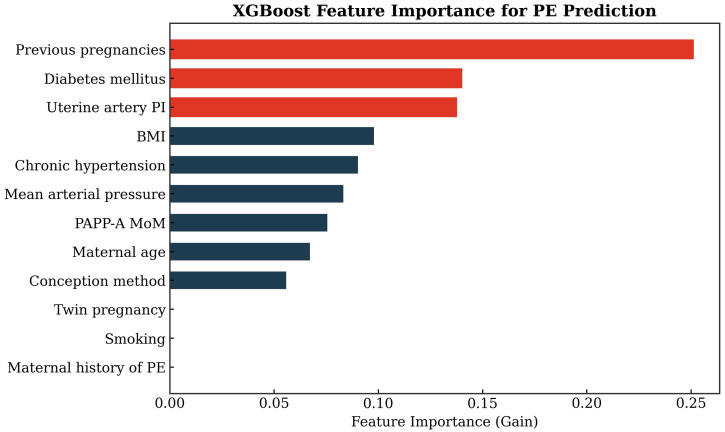
XGBoost feature-importance bar chart for the a posteriori model.

Model interpretability was assessed using SHAP for the a posteriori XGBoost model to characterize which predictors contributed most to the model output. Figure 4 provides a SHAP summary visualization, and Figure 5 presents the corresponding feature importance visualization for the XGBoost model, supporting inference about which predictors the model relied upon most strongly in the a posteriori feature space.

### 3.6. Calibration

**Figure 6:**
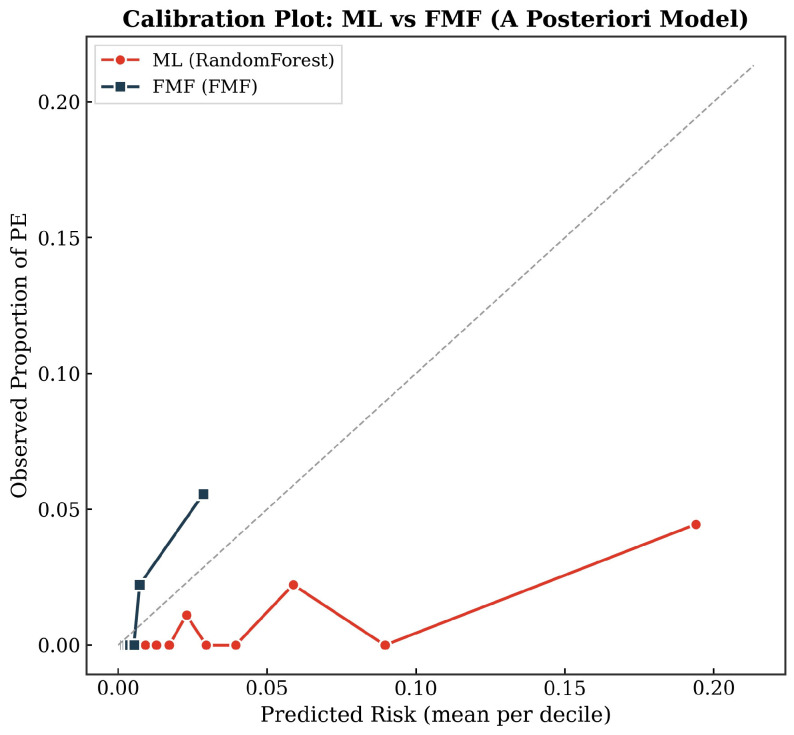
Calibration plot comparing mean predicted risk to observed preeclampsia incidence by decile for ML and FMF a posteriori estimates.

Calibration was examined by comparing observed PE incidence against predicted risk across deciles of predicted risk for both the best ML model and the FMF posterior risk estimate. Figure 6 displays calibration curves, reflecting prior evidence that competing-risks/FMF risk predictions may require recalibration to improve agreement between predicted and observed risks in external cohorts.

### 3.7. Classification at the 1:100 Clinical Threshold and Reclassification

At the clinically used 1:100 risk threshold, the a posteriori FMF model demonstrated a favorable balance of sensitivity (71.4%) and specificity (91.7%), with a screen-positive rate of 8.8%. By contrast, the a posteriori ML model (random forest) achieved 100% sensitivity but at a specificity of 18.0% and a screen-positive rate of 82.1% (Table 4).

At a risk threshold of 1:100, the categorical NRI for the a priori model was +0.11, driven by upward reclassification of events (NRI-events +0.25) that was partially offset by adverse reclassification of non-events (NRI-non-events −0.13)—Table 5. The integrated discrimination improvement (IDI) was negative (−0.010), indicating the FMF model maintained superior overall discrimination. For the a posteriori model, the categorical NRI was −0.45, reflecting excessive upward reclassification of non-events by the ML model (NRI-non-events −0.74), though the IDI was positive (+0.09).

### 3.8. Sensitivity Analysis with Aspirin-Treated Pregnancies Included

Inclusion of the 211 aspirin-treated women did not significantly alter the findings. The a priori ML model (logistic regression) achieved an AUC of 0.79 vs. FMF 0.83 (DeLong *p* = 0.46); the a posteriori ML model (logistic regression) achieved an AUC of 0.87 vs. FMF 0.90 (DeLong *p* = 0.44).

**Figure 7:**
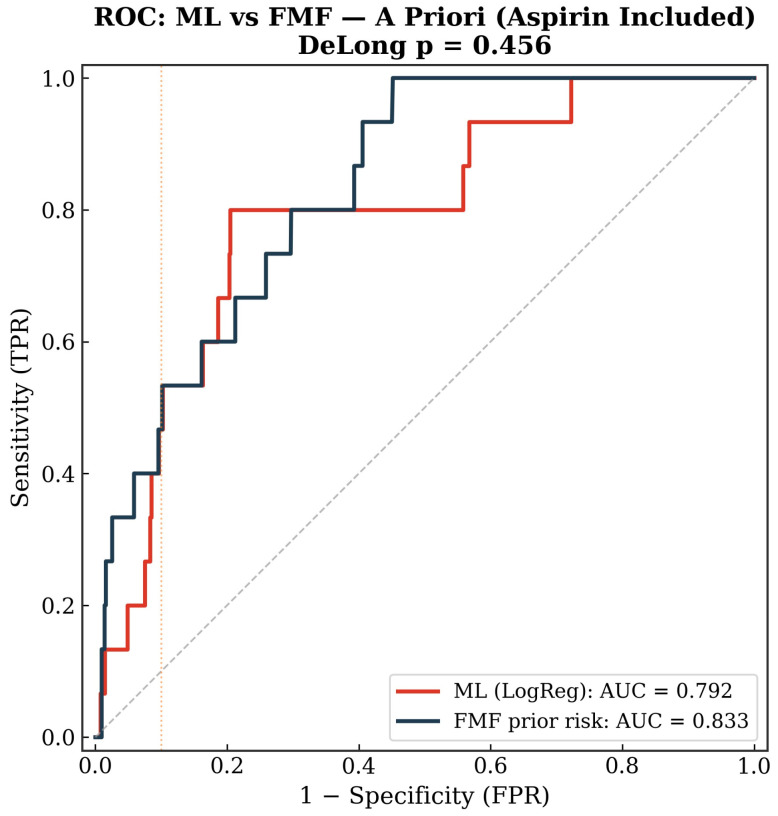
ROC curves for the a priori setting with aspirin-treated pregnancies included.

**Figure 8:**
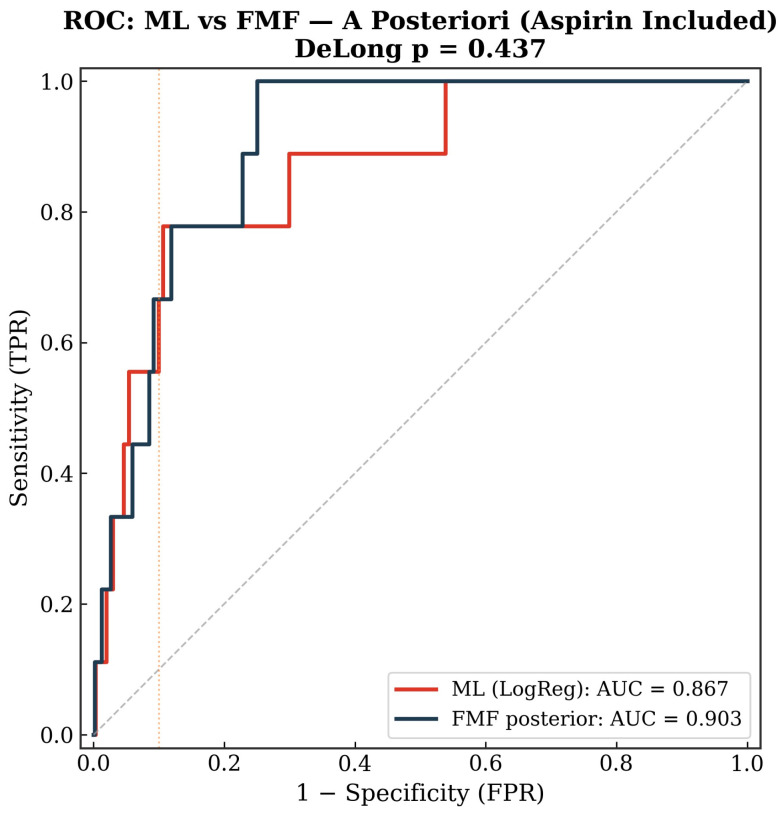
ROC curves for the a posteriori setting with aspirin-treated pregnancies included.

Detection rates at 10% FPR were identical between ML and FMF in both scenarios (46.7% a priori; 66.7% a posteriori), suggesting that the inclusion of aspirin-treated patients, in whom the intervention may have attenuated the preeclampsia phenotype, did not introduce meaningful bias. Figure 7 and Figure 8 show ROC comparisons in a priori and a posteriori settings with aspirin-treated pregnancies included.

### 3.9. Classification at the 1:100 Clinical Threshold

In the cohort that included aspirin-treated pregnancies, at the 1:100 threshold, the FMF posterior model and logistic regression showed similar sensitivity (77.8%), but the FMF model achieved higher specificity (85.5% vs. 82.1%) and a better overall balance of performance, as reflected by a higher F1-score (Table 6).

In the a priori setting, logistic regression achieved the highest sensitivity (80.0%), outperforming the FMF prior model (40.0%), but at the cost of lower specificity. The FMF model demonstrated higher specificity (91.3%) and a slightly higher F1-score, reflecting fewer false positives (Table 5).

### 3.10. Sensitivity Analysis with Random Undersampling

To interrogate the influence of severe class imbalance on ML performance, random undersampling analyses were conducted at PE:non-PE ratios of 1:5 and 1:10, with repeated sampling and evaluation to provide stable median AUC estimates (Table 7). Undersampling experiments at 1:5 and 1:10 PE-to-non-PE ratios produced results consistent with the primary analysis. FMF model consistently outperformed ML models: at 1:5 undersampling, the a posteriori FMF AUC was 0.94 (IQR 0.92–0.97) compared with 0.83 for the best ML model (logistic regression).

**Figure 9:**
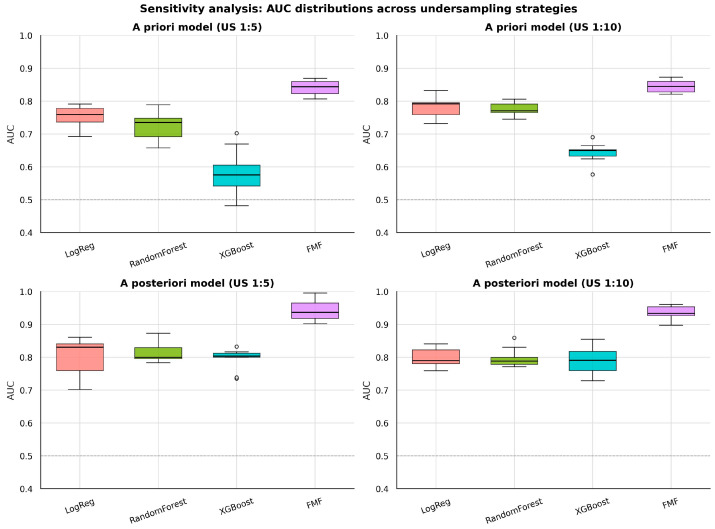
Boxplots of AUC distributions obtained from repeated random undersampling experiments (four panels: a priori 1:5, a priori 1:10, a posteriori 1:5, a posteriori 1:10).

**Figure 10:**
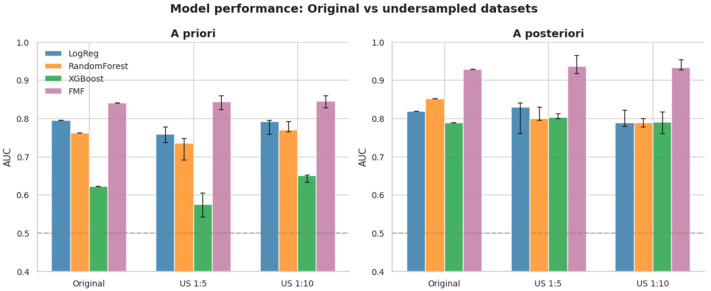
Grouped bar chart comparing AUC across the original analysis and undersampling conditions (US 1:5 and US 1:10) for ML models and FMF risk.

The distributional effects of undersampling on AUC are visualized in Figure 9, with a compact comparison across sampling strategies shown in Figure 10.

### 3.11. Multiple Imputation Sensitivity Analysis

To evaluate the potential impact of missing predictor data, a multiple imputation sensitivity analysis was performed using the full cohort of 1372 participants, including all 12 PE cases (Table 8).

In the a priori analysis, the FMF prior risk model achieved the highest discriminatory performance, with a mean AUC of 0.8409. Among machine learning models, logistic regression showed the best and most stable performance (mean AUC 0.789), followed by random forest (0.752), whereas XGBoost demonstrated substantially lower performance (0.621). Thus, no clear evidence of superiority of the exploratory ML models over the FMF model was observed.

Similarly, in the a posteriori analysis, the FMF posterior risk model retained the highest discrimination (mean AUC 0.8590). Logistic regression and random forest showed comparable performance (mean AUC 0.7607 and 0.7578, respectively), while XGBoost again demonstrated lower and less stable performance (0.6997). Variability across imputations was greater in the a posteriori models, particularly for XGBoost, suggesting reduced model stability when biomarker information was imputed.

Our results indicate that the multiple imputation sensitivity analysis yielded findings consistent with the primary complete-case analyses, with FMF risk models maintaining higher discriminatory performance than the exploratory machine learning approaches.

These results suggest that the main conclusions were not substantially altered by missing biomarker data. However, given the extremely low number of PE cases (n = 12), the substantial degree of biomarker missingness, and the observed variability across imputations, all machine learning findings should be interpreted cautiously and considered exploratory rather than definitive evidence of comparative model performance.

### 3.12. Decision Curve Analysis

**Figure 11:**
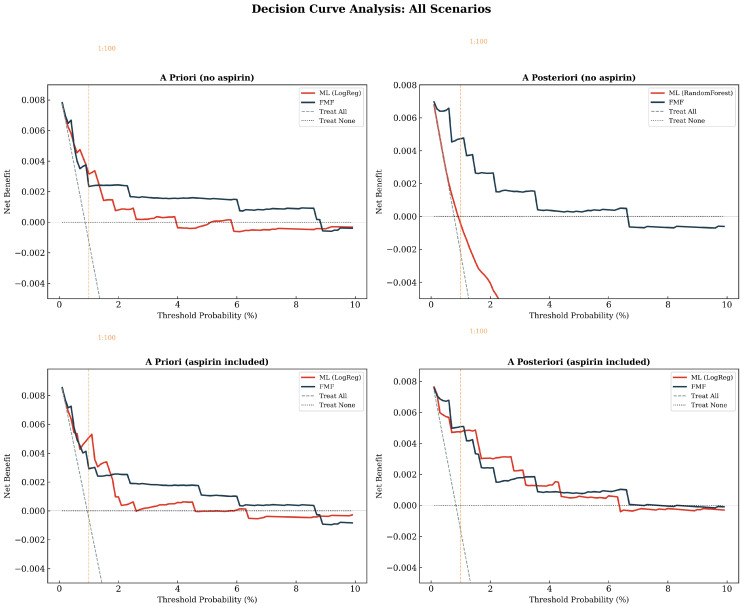
Decision curve analysis comparing net benefit with and without inclusion of aspirin-treated pregnancies.

To assess the clinical utility of each screening strategy beyond discrimination alone, we performed decision curve analysis across a range of clinically relevant risk thresholds (0.5–5%)—Table 9 and Figure 11. Both the ML and FMF models provided positive net benefit across most thresholds and consistently outperformed a “treat all” strategy, confirming that risk-stratified screening is preferable to universal prophylaxis in this low-prevalence population.

However, the FMF algorithm demonstrated superior net benefit in most clinically actionable scenarios. In the a posteriori analysis without aspirin, the setting most relevant to contemporary first-trimester screening, the FMF model exceeded the ML model at every threshold examined (0.5–5%), and the ML model’s net benefit fell below zero at thresholds ≥ 1.0%, indicating that at these thresholds the ML model would cause net harm relative to a strategy of treating no one. This finding is consistent with the calibration deficit observed for the random forest model, whose excessive screen-positive rate (82.1% at the 1:100 threshold) translates directly into an unfavorable balance of true and false positives.

In the a priori (maternal factors only) analysis, the ML model showed a modest advantage over FMF at lower thresholds (0.5–1.0%), where the cost of missing a case is weighted heavily relative to the cost of unnecessary treatment (Figure 10). At higher thresholds (≥2%), where clinicians accept fewer false positives per detected case, the FMF model was consistently superior. This pattern suggests that logistic regression captured slightly more low-risk true positives than the FMF prior model, but at the expense of substantially more false-positive classifications, a trade-off that becomes unfavorable as the threshold rises.

The sensitivity analysis, including aspirin-treated women, did not materially change the pattern. In the a posteriori aspirin-inclusive analysis, the ML model showed marginally higher net benefit at intermediate thresholds (2–3%), but the absolute differences were small (net benefit difference < 0.001) and the FMF model remained superior at the most commonly used clinical thresholds of 0.5–1.0%. At the 1:100 (1%) threshold, the FMF model provided consistently greater net benefit than ML across all four analysis scenarios (a priori, a posteriori, with and without aspirin), with the exception of the a priori analysis without aspirin.

## 4. Discussion

In the present study, the FMF competing-risks algorithm demonstrated consistently superior discriminative performance compared with three ML classifiers across both screening scenarios that we evaluated. In the primary aspirin-excluded cohort, the best-performing ML model using maternal factors only was logistic regression with an AUC of 0.796, whereas the FMF a priori risk achieved an AUC of 0.841, with no statistically significant difference between the two. In the a posteriori setting, the best ML model was random forest with AUC 0.844, while the FMF posterior risk reached AUC 0.929, with a borderline *p*-value of 0.087.

The performance gap widened after adding biomarkers (AUC difference 0.045 a priori to 0.085 a posteriori), and the detection rate at 10% FPR consistently favored FMF (50.0% vs. 33.3% a priori; 71.4% vs. 57.1% a posteriori). These patterns remained directionally stable in sensitivity analyses that included aspirin-treated pregnancies and in repeated random undersampling experiments at PE:non-PE ratios of 1:5 and 1:10.

The undersampling sensitivity analyses may have practical clinical implications. Across different class-imbalance scenarios, the FMF algorithm generally maintained numerically higher and more stable discriminatory performance than the exploratory ML models, suggesting greater robustness in this low-prevalence real-world screening setting.

Our findings align with the broader external-validation literature showing that FMF frequently matches or outperforms alternative statistical or ML models when evaluated head-to-head using comparable predictors and endpoints, and that performance differences can remain even after ML retraining or adjustment of biochemical assays.

Our results can be interpreted through the lens of the way competing-risks screening is constructed, particularly in a low-event-rate cohort. With only 12 PE events in the a priori cohort and seven PE events in the a posteriori cohort, the ML algorithms faced a fundamental information bottleneck in estimating stable decision boundaries from the available positive examples. Expressed as events-per-variable, the effective EPV was approximately 12/9 = 1.3 for the a priori feature set and 7/12 = 0.58 for the a posteriori feature set. On the other hand, the FMF approach is explicitly designed as a Bayes theorem–based risk-updating framework that combines a prior distribution derived from maternal factors with likelihood functions derived from biomarkers to generate individualized posterior risks. The widening of the AUC gap in our cohort after adding biomarkers therefore plausibly reflects the FMF model’s ability to extract risk information from biomarkers through parameterized likelihoods that are stable across cohorts, whereas ML models in our setting must infer those relationships de novo from very few events.

Among the three ML algorithms evaluated, logistic regression demonstrated the most robust performance in the aspirin-excluded a priori screening scenario, while random forest was the most accurate in the a posteriori scenario, and XGBoost was consistently the most unstable. This ordering is noteworthy because much of the ML obstetrics literature reported strong performance for boosting methods (including XGBoost) and for ensemble learners in larger datasets, sometimes showing AUCs that exceed 0.90 when complex biomarker panels and large event counts were available [15,16,17].

A temporally validated cohort study on pre-eclampsia prediction using routinely collected maternal characteristics reported that XGBoost and logistic regression had similar discrimination performance, with AUCs of 0.75 (95% CI 0.73–0.78) and 0.76 (95% CI 0.74–0.78), respectively, while random forest performed worse with an AUC of 0.71 (95% CI 0.69–0.74). The logistic regression model showed near-perfect calibration with a slope of 1.02 (95% CI 0.92–1.12), whereas XGBoost and random forest had calibration slopes of 1.15 (95% CI 1.03–1.28) and 0.62 (95% CI 0.54–0.70), respectively, illustrating that model ranking depends on the dataset and setting rather than being universal [13].

Two systematic reviews also indicated no superiority of machine learning algorithms over logistic regression in clinical prediction models, emphasizing that improvements depend on study design and data characteristics [18,19]. Also, the literature data indicated that logistic regression tends to show similar calibration performance compared to some machine learning models, even though this aspect is not frequently evaluated [10,20].

In our cohort, the comparatively poor a priori performance and high variance of XGBoost (0.623 ± 0.175) are compatible with the notion that higher-capacity models can be more sensitive to small effective sample sizes, even when conventional regularization is used, and reinforce why a transparent, multi-algorithm comparison is preferable to reporting only the best-performing ML approach.

Our incremental biomarker analysis further indicated which components of first-trimester screening were most informative in this cohort and why complete biomarker capture is central for fair comparisons between modeling paradigms. In the XGBoost incremental series, discrimination increased from AUC 0.62 using maternal factors alone to 0.73 after adding MAP, then to 0.81 after adding UtA-PI, and then decreased to 0.79 after adding PAPP-A, while the analyzable sample dropped to 901 patients due to missingness. The magnitude and direction of these increments are concordant with our observed cohort-level associations, in which MAP was significantly higher in PE than non-PE pregnancies and UtA-PI and PAPP-A showed trends in expected directions.

These observations are consistent with the literature demonstrating that combined screening by maternal factors, uterine artery pulsatility index, mean arterial pressure, and placental growth factor achieves substantially higher detection rates than maternal factors alone at fixed false-positive rates, and that such combined approaches have very high discrimination in large cohorts when biomarkers are appropriately incorporated [4,8].

To test whether extreme class imbalance could explain the inferior ML performance relative to FMF models, we conducted a structured random undersampling sensitivity analysis that increased apparent PE prevalence while preserving the positive class size, and the results indicated that undersampling did not improve ML performance relative to FMF in both a priori and a posteriori settings, while the FMF AUC remained stable across sampling strategies.

This pattern resonates with the prior literature that combines resampling strategies such as SMOTE and random undersampling to optimize classifier performance. For example, SMOTE combined with XGBoost consistently improved F1 scores and showed robust performance across imbalance levels, whereas random undersampling often reduced computational burden but did not necessarily enhance predictive accuracy [21]. Other studies found that hybrid methods such as SMOTE-ENN outperform SMOTE alone by generating synthetic samples while removing noisy data, leading to more stable and reliable models [22,23]. However, undersampling techniques tend to have higher variability in results and may not consistently improve discrimination metrics like AUC compared to oversampling methods, which generally provide more stable improvements [24].

Reclassification metrics in our cohort further supported the conclusion that, even when ML increased sensitivity at a given FMF-oriented threshold, it often did so at the cost of adverse reclassification among non-events. The practical implication is that, in a screening setting in which most pregnancies are unaffected, non-event misclassification can dominate program burden and clinical consequences. In the broader screening literature, this trade-off is widely recognized: some external FMF evaluations in high-risk cohorts report higher specificity with a drawback of lower sensitivity, whereas other cohorts achieve stronger detection at fixed false-positive rates, highlighting that both the baseline algorithm and its local calibration and cutoffs matter for the specificity–sensitivity balance [4,25,26]. Our NRI/IDI findings therefore complement the AUC comparisons by making explicit that, at least at the 1:100 cut-off, the ML models did not provide a favorable net reclassification of the much larger non-event group, even when they improved event capture in the small PE subgroup.

Decision curve analysis in our cohort provided the most direct evidence that the FMF approach retained superior clinical utility across plausible decision thresholds, particularly in the a posteriori scenario where the classification trade-offs were most extreme. FMF provided greater net benefit in three of four scenarios examined across the aspirin-excluded and aspirin-included analyses. The interpretive value of DCA in screening research is illustrated by external validations of FMF-type models in which DCA showed net clinical benefit across commonly used threshold ranges (e.g., 1–12%) [6,27], reinforcing the centrality of decision-analytic evaluation when comparing models that might have similar discrimination but different calibration and threshold behavior. In our study, the negative net benefit for the a posteriori ML model at thresholds ≥ 1% is coherent with its extremely low specificity (18.0%) and high screen-positive rate (82.1%) at the 1:100 threshold, because in DCA the harm of false positives is weighted increasingly as threshold probability increases. In other words, even if a model achieves high sensitivity, it can be clinically counterproductive if it generates too many false positives relative to the acceptable trade-off implied by a given clinical threshold.

Our calibration assessment, although necessarily limited by the small number of PE events, suggested that FMF was better calibrated than the ML model in the a posteriori setting, which is consistent with the broader validation literature in which calibration frequently limits transportability of both FMF and ML models across populations [14,27].

Interpretability analyses in our cohort, based on SHAP/feature importance for the a posteriori XGBoost model, suggested that the model relied most heavily on obstetric history, MAP and UtA-PI rather than on the sparse biochemical information available, and these patterns are consistent with both the FMF structure and many ML PE studies [4,28]. In that context, our SHAP pattern can be seen as a model-based reflection of the same physiologic pathways that the FMF competing-risks model encodes explicitly via biomarker likelihoods rather than learning implicitly from limited events.

When we included aspirin-treated pregnancies, the a priori comparison and the a posteriori comparison remained similar, with detection rates at 10% FPR identical between ML and FMF (46.7% a priori; 66.7% a posteriori). These findings suggest that our primary aspirin-excluded analysis did not derive its conclusions solely from treatment-related distortion in the risk–outcome relationship. This interpretation is consistent with external FMF research showing that accounting for aspirin prophylaxis can alter detection rates and PPV and slightly change false-positive rates, and with other cohorts noting that observed performance metrics can already be influenced by aspirin prophylaxis among high-risk women identified through screening programs [29,30]. In our data, the stability of the ML–FMF ranking across aspirin-excluded and aspirin-included analyses supports the conclusion that, in low-events cohorts, FMF risk scores remained the stronger discriminator and the more clinically useful tool across common threshold ranges, even when treatment patterns were incorporated into the analytic dataset.

First-trimester combined screening for preeclampsia is increasingly used as a general tool for stratifying placental and uteroplacental risk, rather than solely to identify candidates for aspirin prophylaxis against preterm preeclampsia [31].

Some literature data showed that women classified as high-risk for preterm preeclampsia in the first trimester have a significantly higher hazard of spontaneous preterm and even term birth despite never developing clinical preeclampsia, suggesting that the preeclampsia risk score captures a broader uteroplacental vulnerability relevant to the timing of spontaneous labor [32,33].

Because the same maternal, Doppler, and biochemical markers also predict fetal growth restriction and small-for-gestational-age birth, first-trimester preeclampsia screening can inform intrapartum surveillance strategies for intrapartum fetal compromise, linking early risk assessment to decisions about intensity of monitoring and timing and mode of delivery [34,35].

Several methodological strengths of our study enhance the credibility of these findings and should be emphasized when positioning our results against heterogeneous literature. We performed a direct head-to-head comparison of three ML algorithms and the FMF risk estimates on the same individuals in both an a priori (maternal factors only) and an a posteriori (maternal plus biomarkers) setting, thereby minimizing confounding due to differing cohorts or outcome definitions. We employed repeated stratified cross-validation (5-fold with 10 repeats) and aggregated out-of-fold probabilities to reduce bias. Beyond discrimination, we reported a detection rate at 10% FPR, threshold performance at 1:100, calibration assessment, DCA, and interpretability analyses, reflecting the multifaceted model evaluation that is increasingly recommended in prediction research. Additionally, our explicit undersampling sensitivity analysis directly tested whether class imbalance could explain the ML–FMF gap, addressing a common methodological critique and linking our findings to prior ML studies that use combined resampling strategies such as SMOTE and random undersampling to improve classifier performance, while demonstrating that such strategies cannot substitute for additional events in small-outcome cohorts.

Our study also has limitations that are inseparable from the observed performance patterns, and that should temper any inference about the absolute superiority of one modeling paradigm over another in different settings. The most consequential limitation is the very low number of PE events: 12 cases in the a priori analysis and seven cases in the complete-case a posteriori analysis, which necessarily yields wide uncertainty around performance metrics and increases the risk that model comparisons are influenced by a small number of influential cases. This restricted statistical power increased the risk of model instability and overfitting, and resulted in relatively wide confidence intervals around several performance estimates. Consequently, all machine learning analyses should be interpreted as exploratory rather than definitive comparisons with the established FMF competing-risks algorithm. Although repeated cross-validation, out-of-fold prediction procedures, and multiple sensitivity analyses were performed to improve robustness, these approaches cannot fully compensate for the limitations imposed by the small event count. Therefore, larger multicenter cohorts with substantially higher numbers of PE cases will be necessary to enable reliable external validation and more robust assessment of ML-based screening approaches.

Second, the study is single-center and Romanian, and numerous external validations demonstrate that FMF performance and calibration can differ materially by population, requiring local correction of biomarker medians or analyzers and, in some contexts, recalibration or population-specific cutoffs; such population dependence is also relevant for ML models, which can be sensitive to measurement distributions and missingness patterns.

Third, the broader prediction literature cautions that even models with good discrimination may have low PPV in the most applicable cohorts and that calibration is frequently not reported, indicating that external validation remains necessary before clinical translation of any ML alternative to an established screening algorithm.

The present findings do not support the replacement of the FMF algorithm by ML approaches in current first-trimester PE screening practice. Rather, the results suggest that established FMF competing-risks models remain robust and clinically reliable in low-prevalence real-world settings. Nevertheless, exploratory ML approaches may still hold future potential, particularly if developed and externally validated in substantially larger multicenter datasets with higher event counts and broader population heterogeneity.

Future work should focus on overcoming current limitations while combining the strengths of FMF and machine learning approaches and should include large multicenter studies with sufficient preeclampsia cases to enable robust training, especially for early-onset and preterm forms, and improving biomarker completeness and standardization. Hybrid strategies, such as integrating FMF risk scores into ML models or using ML for recalibration, should also be explored to reduce false positives and improve clinical usefulness.

Moreover, future evaluations should jointly assess discrimination, calibration, and clinical utility, recognizing that in low-prevalence settings, meaningful improvements are more likely to come from reducing false positives and increasing net clinical benefit rather than small gains in AUC alone.

## 5. Conclusions

Overall, the FMF competing-risks model demonstrated consistently superior discrimination and clinical utility compared with the evaluated ML algorithms in our first-trimester screening setting, and our resampling-based sensitivity analyses support the interpretation that ML underperformance was not primarily driven by class imbalance but by limited event counts and the absence of FMF’s domain-encoded Bayesian structure.

These findings are consistent with external evidence that FMF is a widely used screening standard and that algorithm performance depends strongly on population-specific calibration, biomarker measurement standardization, and clinically relevant operating points rather than on AUC alone.

In low-prevalence settings with constrained biomarker availability, the FMF algorithm remains an appropriate reference approach, while future ML development should emphasize external validation, assay-aware preprocessing, and evaluation of calibration and net benefit to justify adoption.

## Figures and Tables

**Table 1:** Baseline Characteristics of the Study Population by Preeclampsia Status (Aspirin-Excluded Cohort, n = 1372).

Variable	PE (n = 12)	Non-PE (n = 1360)	*p*-Value
Age, years, median (IQR)	31.3 (25.2–34.7)	29.9 (27.3–33.3)	0.93
BMI, kg/m^2^, median (IQR)	24.7 (23.5–27.9)	22.1 (20.3–24.7)	0.004
MAP, mmHg, median (IQR)	93.4 (92.0–94.2)	88.7 (83.8–92.9)	0.016
UtA-PI, median (IQR)	1.9 (1.6–2.0)	1.6 (1.3–1.9)	0.081
PAPP-A MoM, median (IQR)	0.5 (0.3–1.0)	1.0 (0.6–1.4)	0.091
Chronic hypertension, n (%)	3 (25.0)	20 (1.5)	<0.001
Diabetes mellitus, n (%)	1 (8.3)	5 (0.4)	0.051
Smoking in pregnancy, n (%)	0 (0)	30 (2.2)	1.0
Maternal PE history, n (%)	1 (8.3)	16 (1.2)	0.120
Twin pregnancy, n (%)	0 (0)	10 (0.7)	1.0
Nulliparity, n (%)	5 (41.7)	749 (55.1)	0.396

Legend: BMI—Body Mass Index; MAP—Mean Arterial Pressure; UtA-PI—Uterine Artery Pulsatility Index; PAPP-A—Pregnancy-Associated Plasma Protein A; MoM—Multiple of the Median; PE—Preeclampsia; IQR—Interquartile Range.

**Table 2:** Comparison between participants included and excluded from the a posteriori complete-case analysis.

Variable	Included Complete Cases (n = 901)	Excluded Cases (n = 471)	*p*-Value
Maternal age (years)	30.20 (27.60–33.40)	29.50 (26.85–33.15)	0.0427
BMI (kg/m^2^)	22.15 (20.31–24.86)	22.06 (20.32–24.61)	0.5694
MAP (mmHg)	89.00 (84.16–93.08)	87.84 (82.70–92.50)	0.0305
UtA-PI	1.57 (1.32–1.89)	1.66 (1.39–1.96)	0.0014
PAPP-A MoM	0.97 (0.65–1.44)	0.66 (0.39–1.23)	0.0275
FMF prior risk	0.00 (0.00–0.01)	0.00 (0.00–0.01)	0.0010
FMF posterior risk	0.00 (0.00–0.01)	0.00 (0.00–0.01)	<0.001
Preeclampsia outcome	7/901 (0.8%)	5/471 (1.1%)	0.8163
Smoking during pregnancy	81/901 (9.0%)	30/471 (6.4%)	0.1128
Diabetes mellitus	3/901 (0.3%)	3/471 (0.6%)	0.4190
Chronic hypertension	16/901 (1.8%)	8/471 (1.7%)	1.0000
Maternal history of PE	2/901 (0.2%)	2/471 (0.4%)	0.6107
Twin pregnancy	0/901 (0.0%)	0/471 (0.0%)	1.0000

Legend: BMI—Body Mass Index; MAP—Mean Arterial Pressure; UtA-PI—Uterine Artery Pulsatility Index; PAPP-A—Pregnancy-Associated Plasma Protein A; MoM—Multiple of the Median; PE—Preeclampsia.

**Table 3:** Performance of ML models and the FMF algorithm, excluding aspirin-treated pregnancies.

Scenario	CV AUC LR	CV AUC RF	CV AUC XGBoost	Final ML AUC (95% CI)	FMF AUC (95% CI)	DeLong *p*	ML DR at 10% FPR	FMF DR at 10% FPR
A priori	0.7958 ± 0.1112	0.7632 ± 0.0861	0.6226 ± 0.1752	0.7897 (0.6537–0.8962)	0.8409 (0.7487–0.9233)	0.3487	33.3%	50.0%
A posteriori	0.8196 ± 0.1902	0.8519 ± 0.1366	0.7885 ± 0.1434	0.8442 (0.6878–0.9616)	0.9292 (0.8721–0.9751)	0.0866	57.1%	71.4%

Legend: CV—Cross-Validation; AUC—Area Under the Curve; LR—Logistic Regression; RF—Random Forest; XGBoost—Extreme Gradient Boosting; ML—Machine Learning; FMF—Fetal Medicine Foundation; DR—Detection Rate; FPR—False Positive Rate; DeLong *p*—DeLong test *p*-value.

**Table 4:** Classification metrics at the FMF threshold of 1:100 (risk ≥ 0.01), excluding aspirin-treated pregnancies.

A Priori (1372 Patients)										
Model	TP	FP	FN	TN	Sensitivity	Specificity	PPV	NPV	F1-score	Accuracy
Logistic regression	7	265	5	1095	58.3 (32.0–80.7)	80.5 (78.3–82.5)	2.6 (1.3–5.2)	99.5 (98.9–99.8)	0.0493	80.3 (78.1–82.3)
Random forest	12	1355	0	5	100.0 (75.8–100.0)	0.4 (0.2–0.9)	0.9 (0.5–1.5)	100.0 (56.6–100.0)	0.0174	1.2 (0.8–2.0)
XGBoost	5	313	7	1047	41.7 (19.3–68.1)	77.0 (74.7–79.1)	1.6 (0.7–3.6)	99.3 (98.6–99.7)	0.0303	76.7 (74.4–78.8)
FMF (prior_risk)	4	79	8	1281	33.3 (13.8–60.9)	94.2 (92.8–95.3)	4.8 (1.9–11.8)	99.4 (98.8–99.7)	0.0842	93.7 (92.2–94.8)
A Posteriori (901 Patients)										
Model	TP	FP	FN	TN	Sensitivity	Specificity	PPV	NPV	F1-score	Accuracy
Logistic regression	5	158	2	736	71.4 (35.9–91.8)	82.3 (79.7–84.7)	3.1 (1.3–7.0)	99.7 (99.0–99.9)	0.0588	82.2 (79.6–84.6)
Random forest	7	733	0	161	100.0 (64.6–100.0)	18.0 (15.6–20.7)	0.9 (0.5–1.9)	100.0 (97.7–100.0)	0.0187	18.6 (16.2–21.3)
XGBoost	4	168	3	726	57.1 (25.1–84.2)	81.2 (78.5–83.6)	2.3 (0.9–5.8)	99.6 (98.8–99.9)	0.0447	81.0 (78.3–83.4)
FMF (posterior)	5	74	2	820	71.4 (35.9–91.8)	91.7 (89.7–93.4)	6.3 (2.7–14.0)	99.8 (99.1–99.9)	0.1163	91.6 (89.6–93.2)

Legend: PPV—Positive Predictive Value; NPV—Negative Predictive Value; FMF—Fetal Medicine Foundation; TP—true positive; FP—false positive; FN—false negative; TN—true negative; XGBoost—Extreme Gradient Boosting.

**Table 5:** Comparative reclassification analysis between ML models and FMF risk estimates: bootstrap AUC, NRI, and IDI.

Scenario	ML AUC (95% CI)	FMF AUC (95% CI)	Categorical NRI	Event NRI	Non-Event NRI	Continuous NRI	IDI
A priori	0.789 (0.652–0.902)	0.840 (0.748–0.925)	0.113	0.250	−0.136	−0.672	−0.010
A posteriori	0.846 (0.700–0.967)	0.9298 (0.880–0.976)	−0.451	0.285	−0.737	0.006	0.091

Legend: ML—Machine Learning; AUC—Area Under the Curve; CI—Confidence Interval; FMF—Fetal Medicine Foundation; NRI—Net Reclassification Improvement; IDI—Integrated Discrimination Improvement.

**Table 6:** Classification metrics at the FMF threshold of 1:100 (risk ≥ 0.01), including aspirin-treated pregnancies.

A Priori (1583 Patients)										
Model	TP	FP	FN	TN	Sensitivity	Specificity	PPV	NPV	F1-score	Accuracy
Logistic regression	12	392	3	1176	80.0 (54.8–93.0)	75.0 (72.8–77.1)	3.0 (1.7–5.1)	99.7 (99.3–99.9)	0.0573	75.0 (72.9–77.1)
Random forest	15	1565	0	3	100.0 (79.6–100.0)	0.2 (0.1–0.6)	0.9 (0.6–1.6)	100.0 (43.9–100.0)	0.0188	1.1 (0.7–1.8)
XGBoost	10	384	5	1184	66.7 (41.7–84.8)	75.5 (73.3–77.6)	2.5 (1.4–4.6)	99.6 (99.0–99.8)	0.0489	75.4 (73.3–77.5)
FMF (prior risk)	6	136	9	1432	40.0 (19.8–64.3)	91.3 (89.8–92.6)	4.2 (2.0–8.9)	99.4 (98.8–99.7)	0.0764	90.8 (89.3–92.2)
A Posteriori (1072 Patients)										
Model	TP	FP	FN	TN	Sensitivity	Specificity	PPV	NPV	F1-score	Accuracy
Logistic regression	7	190	2	873	77.8 (45.3–93.7)	82.1 (79.7–84.3)	3.6 (1.7–7.2)	99.8 (99.2–99.9)	0.0680	82.1 (79.7–84.3)
Random forest	9	905	0	158	100.0 (70.1–100.0)	14.9 (12.9–17.1)	1.0 (0.5–1.9)	100.0 (97.6–100.0)	0.0195	15.6 (13.5–17.9)
XGBoost	6	213	3	850	66.7 (35.4–87.9)	80.0 (77.5–82.3)	2.7 (1.3–5.9)	99.6 (99.0–99.9)	0.0526	79.9 (77.3–82.1)
FMF (posterior risk)	7	154	2	909	77.8 (45.3–93.7)	85.5 (83.3–87.5)	4.3 (2.1–8.7)	99.8 (99.2–99.9)	0.0824	85.5 (83.2–87.4)

Legend: PPV—Positive Predictive Value; NPV—Negative Predictive Value; FMF—Fetal Medicine Foundation; TP—true positive; FP—false positive; FN—false negative; TN—true negative; XGBoost—Extreme Gradient Boosting.

**Table 7:** Sensitivity Analysis: Random Undersampling Results (Median AUC and IQR Across 10 Repetitions).

Scenario	Sampling Strategy	Best ML Model and AUC	FMF AUC	Interval Type	DeLong *p*	PE Prevalence
A priori	Original (no undersampling)	0.790 (0.654–0.896) [LogReg]	0.841 (0.749–0.923)	95% CI	0.349	0.9%
A posteriori	Original (no undersampling)	0.844 (0.688–0.962) [RandomForest]	0.929 (0.872–0.975)	95% CI	0.087	0.8%
A priori	US 1:5	0.759 (0.737–0.778) [LogReg]	0.844 (0.823–0.860)	IQR	0.163	16.7%
A priori	US 1:10	0.792 (0.759–0.795) [LogReg]	0.845 (0.828–0.860)	IQR	0.187	9.1%
A posteriori	US 1:5	0.831 (0.760–0.841) [LogReg]	0.937 (0.918–0.965)	IQR	0.104	16.7%
A posteriori	US 1:10	0.793 (0.777–0.813) [RandomForest]	0.934 (0.927–0.954)	IQR	0.116	9.1%

Legend: ML—Machine Learning; AUC—Area Under the Curve; FMF—Fetal Medicine Foundation; DeLong *p*—DeLong test *p*-value; PE—Preeclampsia; US—Under-sampling; LogReg—Logistic Regression; IQR—Interquartile Range.

**Table 8:** Multiple imputation sensitivity analysis for missing predictor data (1372 patients, 12 PE cases).

Analysis	Model	Mean AUC (95% CI)
A priori	Logistic regression	0.789 (0.769–0.810)
A priori	Random forest	0.752 (0.740–0.764)
A priori	XGBoost	0.62 (0.591–0.652)
A priori	FMF (prior risk)	0.841 (0.841–0.841)
A posteriori	Logistic regression	0.760 (0.687–0.834)
A posteriori	Random forest	0.757 (0.676–0.840)
A posteriori	XGBoost	0.699 (0.578–0.822)
A posteriori	FMF (posterior risk)	0.859 (0.859–0.859)

Legend: ML—Machine Learning; AUC—Area Under the Curve; FMF—Fetal Medicine Foundation; XGBoost—Extreme Gradient Boosting.

**Table 9:** Net benefit at clinically relevant thresholds.

A Priori (No Aspirin)			
Threshold	NB ML	NB FMF	NB Treat All
0.5%	0.005106	0.005018	0.003765
1.0%	0.003151	0.002334	−0.001266
2.0%	0.000803	0.002439	−0.011483
3.0%	0.000195	0.001623	−0.021911
5.0%	−0.000153	0.001534	−0.043425
A posteriori (no aspirin)			
Threshold	NB ML	NB FMF	NB Treat All
0.5%	0.002833	0.006447	0.002783
1.0%	−0.000448	0.004720	−0.002253
2.0%	−0.004054	0.002627	−0.012480
3.0%	−0.006591	0.001499	−0.022918
5.0%	−0.008178	0.000292	−0.044454
A priori (aspirin included)			
Threshold	NB ML	NB FMF	NB Treat All
0.5%	0.005419	0.005793	0.004498
1.0%	0.005079	0.002922	−0.000530
2.0%	0.000967	0.002553	−0.010739
3.0%	0.000208	0.001843	−0.021159
5.0%	−0.000033	0.001064	−0.042657
A posteriori (aspirin included)			
Threshold	NB ML	NB FMF	NB Treat All
0.5%	0.005728	0.006713	0.003413
1.0%	0.004740	0.005079	−0.001621
2.0%	0.003046	0.002418	−0.011841
3.0%	0.002260	0.001789	−0.022273
5.0%	0.000589	0.000786	−0.043794

Legend: NB—Net Benefit; ML—Machine Learning; FMF—Fetal Medicine Foundation.

## Data Availability

The datasets are available from the corresponding authors upon a reasonable request due to local policies.
